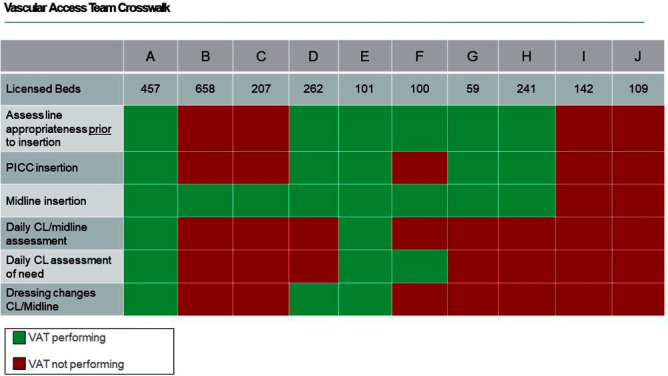# Impact of Vascular Access Teams on Central Line Associated Bloodstream Infections

**DOI:** 10.1017/ash.2024.109

**Published:** 2024-09-16

**Authors:** Shelley Kester, Katie Passaretti, Mindy Sampson, Anupama Neelakanta, Robert Rose, David Tincopa, Johnna Parsons, Audrey Weaver, Abby Avery, Barbi Mills, Candice Dickerson

**Affiliations:** Atrium Health; Stanford University; Carolinas HealthCare System; Atrium Health Cabarrus; Vascular Access RN

## Abstract

**Background:** During the COVID-19 pandemic, rates of central line bloodstream infections (CLABSI) increased nationally. Studies pre-pandemic showed improved CLABSI rates with implementation of a standardized vascular access team (VAT).[PL1] [PL2] [mi3] Varying VAT resources and coverage existed in our 10 acute care facilities (ACF) prior to and during the pandemic. VAT scope also varied in 1) process for line selection during initial placement, 2) ability to place a peripherally inserted central catheter (PICC), midline or ultrasound-guided peripheral IV in patients with difficult vascular access, 3) ownership of daily assessment of central line (CL) necessity, and 4) routine CL dressing changes. We aimed to define and implement the ideal VAT structure and evaluate the impact on CLABSI standardized infection ratios (SIR) and rates prior to and during the pandemic. **Methods:** A multidisciplinary workgroup including representatives from nursing, infection prevention, and vascular access was formed to understand the current state of VAT responsibilities across all ACFs. The group identified key responsibilities a VAT should conduct to aid in CLABSI prevention. Complete VAT coverage[mi4] was defined as the ability to conduct the identified responsibilities daily. We compared the SIR and CLABSI rates between hospitals who had complete VAT (CVAT) coverage to hospitals with incomplete VAT (IVAT) coverage. Given this work occurred during the pandemic, we further stratified our analysis based on a time frame prior to the pandemic (1/2015 – 12/2019) and intra-pandemic (1/2020 - 12/2022). **Results:** The multidisciplinary team identified 6 key components of complete VAT coverage: Assessment for appropriate line selection prior to insertion, ability to insert PICC and midlines, daily CL and midline care and maintenance assessments, daily assessment of necessity for CL, and weekly dressing changes for CL and midlines[NA5] . A cross walk of VAT scope (Figure 1) was performed in October 2022 which revealed two facilities (A and E) which met CVAT criteria. Pre-pandemic, while IVAT CLABSI rates and SIR were higher than in CVAT units, the difference was not statistically significant. During the pandemic, however, CLABSI rates and SIR were 40-50% higher in IVAT compared to CVAT facilities (Incident Rate Ratio 1.5, 95% CI 1.1-2.0 and SIR Relative Ratio 1.4, 95% CI1.1-1.9 respectively) (Table 1). **Conclusions:** CLABSI rates were lower in facilities with complete VAT coverage prior to and during the COVID-19 pandemic suggesting a highly functioning VAT can aid in preventing CLABSIs, especially when a healthcare system is stressed and resources are limited.

**Figure:**
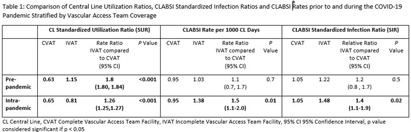

**Figure:**